# Comparison of global extracellular volume (ECV) and late gadolinium enhancement (LGE) to predict the estimated 5 year risk of sudden cardiac death (SCD) in patients with hypertrophic cardiomyopathy (HCM)

**DOI:** 10.1186/1532-429X-18-S1-P127

**Published:** 2016-01-27

**Authors:** Maxim Avanesov, Julius Weinrich, Julia Münch, Lennart Well, Dennis Säring, Kai Müllerleile, Enver Tahir, Monica Patten, Gerhard Adam, Gunnar Lund

**Affiliations:** 1 grid.13648.380000000121803484University Medical Center Hamburg-Eppendorf, Hamburg, Germany; 2 grid.13648.380000000121803484General and Interventional Cardiology, University Heart Center Hamburg, Hamburg, Germany

## Background

HCM is the main cause for SCD in adults and evidence of myocardial fibrosis is associated with poor prognosis. In 2014, a new risk score to predict SCD within 5 years has been established for HCM patients by the European Society of Cardiology (ESC). A score ≥ 6% indicates ICD implantation according to the current ESC recommendations. We analyzed quantitatively global myocardial fibrosis using ECV and LGE measurement in HCM patients. Correlations and ROC curves were performed between LGE, ECV and the SCD risk score to evaluate the value of these quantitative imaging parameters to predict estimated SCD risk in HCM patients.

## Methods

CMR was performed in 62 patients with HCM (53 ± 16 years, 34 men) and 16 healthy volunteers (51,8 ± 9 years, 8 men) using a 1.5T scanner (Achieva,Philips). Post-contrast images were obtained after injection of 0.075 mmol/kg Gd-BOPTA. Two observers independently analyzed fibrosis size on phase-sensitive inversion-recovery (PSIR) LGE-images and on ECV maps. Mean fibrosis size was assessed on 3 short axes obtained at the apex, center and basis of the left ventricle (LV) and expressed in % of LV area (%LV). On LGE images fibrosis was quantified using a threshold method of >2SD above normal appearing myocardium. For ECV a threshold ≥30% was defined, which was >2SD above the mean ECV of 25.8% ± 4.2% assessed from 16 healthy volunteers. The SCD risk score was calculated for each patient. Global LGE and global ECV were correlated with the risk score. ROC Analysis was used to identify optimal thresholds and areas under the curves (AUCs) for a risk score of ≥ 6%. The Youden index was used to depict optimal cutoff values from the ROC curves.

## Results

**Figure 1:**
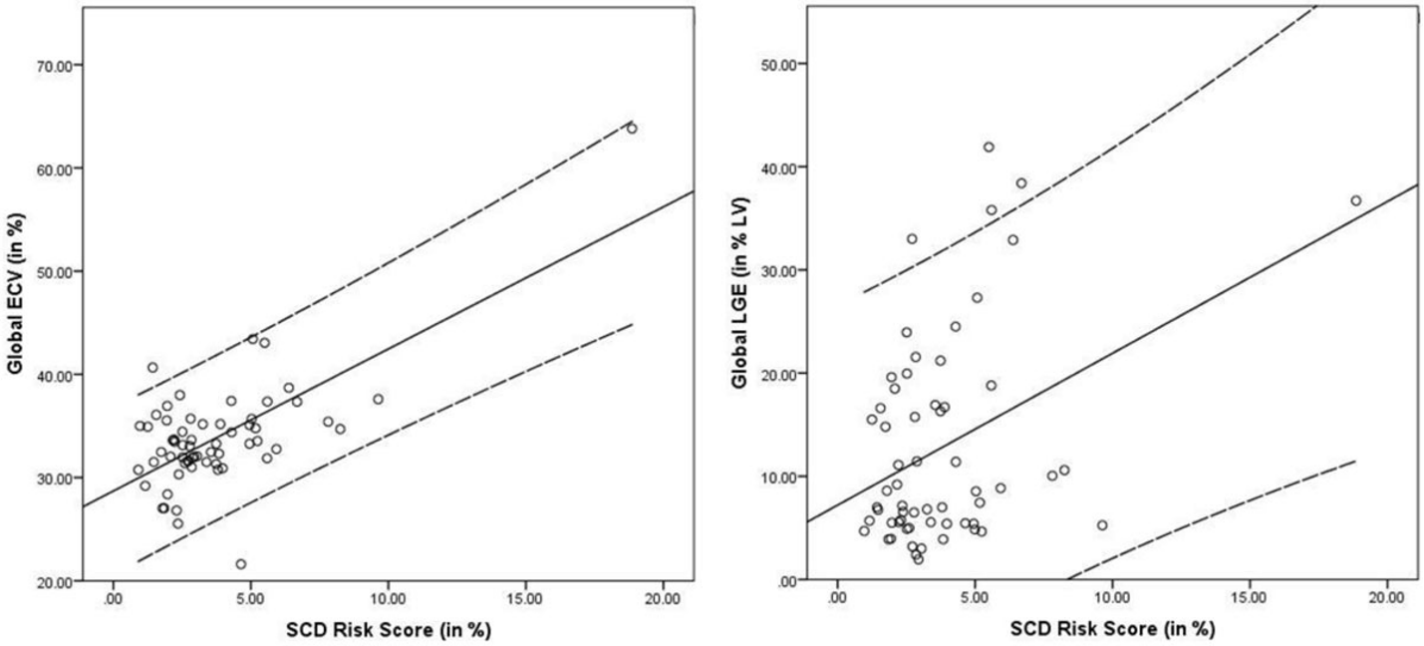
**Correlation of global ECV (R = 0.68, p < 0.01) and global LGE with the SCD Risk Score (R = 0.39, p = 0.08)**. Dashed lines define a 95% confidence interval. Continuous line represents the fitting line.

**Figure 2:**
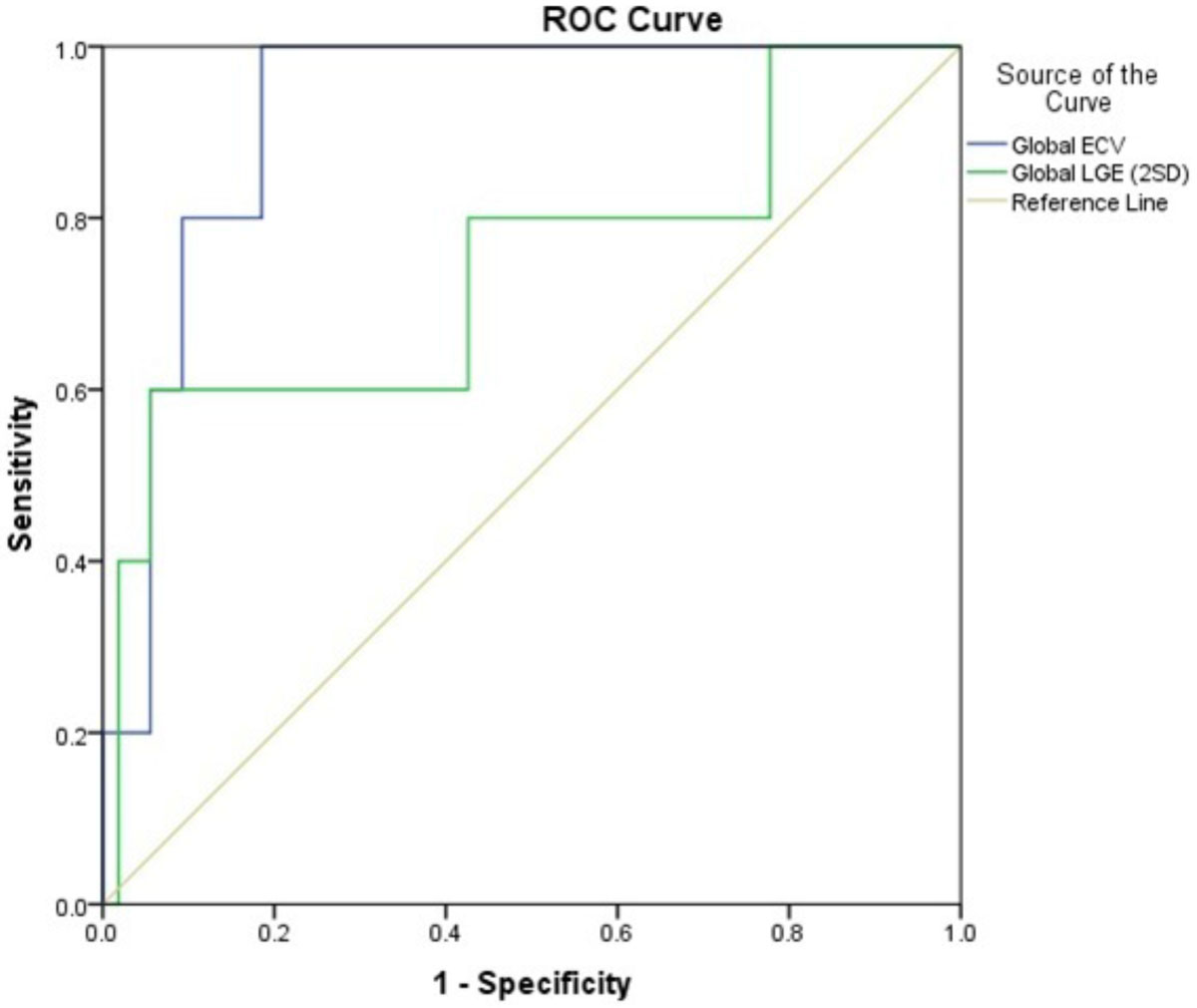
**ROC Curves of global ECV and global LGE for discrimination of HCM patients with a SCD risk score of ≥6% at 5 years**.

LGE was present in 66% of HCM patients. The risk score was not different in patients with and without LGE (p = 0.063), whereas significantly higher scores were present in patients with a global ECV of ≥30% (p = 0.016). A good correlation was found between ECV and the risk score (R = 0.68, p < 0.01) while LGE revealed a lower correlation with the risk score (R= 0.39, p = 0.08 versus ECV; Fig 1). ROC-analysis showed that ECV≥ 34.6% had a better AUC with 0.92, p = 0.002 to predict a risk score of ≥ 6% compared to LGE using a fibrosis size of ≥30.1 %LV as cutoff with an AUC of 0.74, p = 0.07, Fig. 2).

## Conclusions

Global ECV correlated best with the clinical risk score to predict SCD in HCM patients. Furthermore, global ECV ≥ 34.6% had a very good test performance to predict an increased clinical risk score of ≥ 6% indicating its potential value for risk stratification in patients with HCM.

